# Changes in selective pressures associated with human population expansion may explain metabolic and immune related pathways enriched for signatures of positive selection

**DOI:** 10.1186/s12864-016-2783-2

**Published:** 2016-07-21

**Authors:** Alexandra I. Vatsiou, Eric Bazin, Oscar E. Gaggiotti

**Affiliations:** Laboratoire d’Écologie Alpine (LECA), Univesrity Joseph Fourier, 2233 Rue de la Piscine, 38041 Grenoble, Cedex 9, France; Scottish Oceans Institute, East Sands, University of St Andrews, St Andrews, KY16 8LB Scotland UK; Oh no sequences! Research group, Era7Bioinformatics, Plaza de Campo Verde, 3, 18001 Granada, Spain

**Keywords:** Gene set enrichment analysis, Polygenic selection, Immunity, Metabolism

## Abstract

**Background:**

The study of local adaptation processes is a very important research topic in the field of population genomics. There is a particular interest in the study of human populations because they underwent a process of rapid spatial expansion and faced important environmental changes that translated into changes in selective pressures. New mutations may have been selected for in the new environment and previously existing genetic variants may have become detrimental. Immune related genes may have been released from the selective pressure exerted by pathogens in the ancestral environment and new variants may have been positively selected due to pathogens present in the newly colonized habitat. Also, variants that had a selective advantage in past environments may have become deleterious in the modern world due to external stimuli including climatic, dietary and behavioral changes, which could explain the high prevalence of some polygenic diseases such as diabetes and obesity.

**Results:**

We performed an enrichment analysis to identify gene sets enriched for signals of positive selection in humans. We used two genome scan methods, XPCLR and iHS to detect selection using a dense coverage of SNP markers combined with two gene set enrichment approaches. We identified immune related gene sets that could be involved in the protection against pathogens especially in the African population. We also identified the glycolysis & gluconeogenesis gene set, related to metabolism, which supports the thrifty genotype hypothesis invoked to explain the current high prevalence of diseases such as diabetes and obesity. Extending our analysis to the gene level, we found signals for 23 candidate genes linked to metabolic syndrome, 13 of which are new candidates for positive selection.

**Conclusions:**

Our study provides a list of genes and gene sets associated with immunity and metabolic syndrome that are enriched for signals of positive selection in three human populations (Europeans, Africans and Asians). Our results highlight differences in the relative importance of pathogens as drivers of local adaptation in different continents and provide new insights into the evolution and high incidence of metabolic syndrome in modern human populations.

**Electronic supplementary material:**

The online version of this article (doi:10.1186/s12864-016-2783-2) contains supplementary material, which is available to authorized users.

## Background

Migration and colonization of new habitats are a major component of human demographic history. Such processes lead to sharp changes in environmental conditions experienced by individuals (soil, water, climate, and pathogens) with concomitant changes in lifestyle and diet, which in turn alter the phenotypic composition of human populations. Changes in environmental conditions can lead to shifts in selective pressures. Thus, new mutations may be advantageous or previous favorable ones may become deleterious [1, 2].

Diseases caused by deleterious mutations are not expected to persist for many generations or to segregate at high frequency. Yet, the prevalence of metabolic syndrome such as diabetes type 2 and obesity has increased dramatically in recent years, especially in industrialized countries [3, 4]. In principle, natural selection should act to eliminate harmful genetic variants but there are many reasons why this is not always the case. One of the most well-known explanations for the high incidence of metabolic syndrome is the “Thrifty Genotype Hypothesis” [5], which posits that alleles that had a selective advantage in past environments became deleterious in the modern world due to external stimuli including climatic, dietary and behavioral changes. A non-adaptive alternative to this hypothesis is the “Drifty Gene Hypothesis”, which contends that early hominins were subject to predation risks that selected against mutations associated with increased weigh. However, the evolution of social behavior, discovery of fire and use of weapons released more recent Homo species from such selective pressure allowing metabolic disease genes to evolve under neutrality [6]. Also, there are many other possible explanations. For example, a deleterious mutation may be driven to high frequency due to linkage disequilibrium with a neighboring locus/region that is under positive selection [7, 8]. Also, an allele might have pleiotropic effects, for example increased disease risk and protection against a bacterial infection [9].

Another good example of changes in selective pressures exerted on humans during their spatial expansion relates to the pathogens they found in the newly colonized habitats. Infectious diseases caused by pathogens are one of the main threats to human populations and there is an increasing interest in the identification of genomic regions associated with parasite resistance or susceptibility [10, 11]. It is well known that the evolutionary arms race between hosts and pathogens can lead to selective pressures on immune related gene sets [11–16]. Recent studies provide several examples of pathogen-driven selection signatures with important implications in terms of human immunology and infectious disease epidemiology [10]. Thus, it is important to investigate if the application of new statistical genetic approaches can uncover additional examples.

The purpose of the present study is to investigate the extent to which colonization of new habitats by humans represent important changes in selective pressures that may explain human phenotypic variation related to common diseases and immunity. In particular, changes in diet are likely to represent important new selective pressures that may explain the high prevalence of diabetes type 2. Additionally, differences in parasite species prevalent in different habitats may also lead to changes in selective pressures on immunity related genes. Thus, we focus on the impact that environmental changes may have on metabolic syndrome and infectious diseases. Until recently, a substantial understanding of the evolution of such complex diseases has been derived from candidate variants that were revealed either by experiments or gene association studies [17, 18]. There is general agreement that complex diseases are inherently polygenic and, therefore, genome scans based solely on SNP data are not well suited for making inferences about their genetic architecture. Individual variants associated with complex traits tend to be weakly selected and, therefore, only the combined effect on gene sets can be strong enough to reach strict cutoff levels of significance. This limitation can be overcome by combining genome scans of SNP data with gene-set enrichment analyses [19]. This approach detects selection signatures at the gene set level and, therefore, is better adapted to detect polygenic selection. For this reason, we first carry out genome scans of selection (XPCLR [20] and iHS [21]) using HapMap SNP data [22] and then we use their output as input of Gene Set Enrichment Analysis (GSEA-Daub et al. [19] and Gowinda [23]). We mainly focus on the gene set level and we refine it to the gene level whenever needed.

We detected gene sets involved in immunity and host-defense interactions in African. We further examined how natural selection has influenced the prevalence of diseases such as diabetes type 2 and obesity, investigating at both the gene and gene set level. Our results indicate that changes in environmental conditions have lead to shifts in selective pressures on genomic regions associated with adaptation to pathogens and with phenotypes associated to metabolic syndrome.

## Methods

### SNP data

We use SNP data from Hapmap database phase II [22] to test for signatures of positive selection using two genome-scan methods, XPCLR [20] and iHS [21] (the data from HapMap Project are publicly available for unrestricted research use). These methods provide selection scores for each of the SNPs in the dataset, which we later use to perform the enrichment analyses.

The data consists of three populations: Europeans (CEU), Yoruba from Ibadan, Nigeria (YRI), and Han Chinese from Beijing, China and Japanese from Tokyo (CHB + JTP). Recombination rates and genetic coordinates are from assembly NCBI36 [24].

### Genome scan methods

Several methods have been developed to detect signals of positive selection. Fst-based [25] and haplotype homozygosity methods [21, 26] are among the most widely used. Although these methods detect genomic regions under positive selection, they do not necessarily detect the same signals. A sensitivity analysis [27] that compared the performance of many recent methods under complex evolutionary scenarios revealed that the Cross Population Composite Likelihood Ratio test (XPCLR) [20] performs the best under both hard and soft sweep scenarios. However, it lacks power to detect very recent (incomplete) selective sweeps, in which case the integrated Haplotype Score (iHS) [21] performs better. More specifically, iHS has the ability to detect selective sweeps at an early stage but lacks power to detect those at intermediate or late stages while XPCLR exhibits the exact opposite behaviour. Another substantial difference between the two genome-scan methods is the underlying theory that they use to identify candidate regions under positive selection. XPCLR is a population differentiation method, which uses the multilocus allele frequency differentiation between two populations; the objective population (under positive selection) and the reference population (under neutrality). On the other hand, iHS is a haplotype-based method that considers the extent of linkage disequilibrium surrounding the core SNP in an isolated population. Therefore, we chose to combine XPCLR and iHS to maximize the power to detect selection signatures at the SNP level.

#### XPCLR analyses

We carried out XPCLR analyses of all possible population pairs from the Hapmap Phase II dataset. In all of the comparisons, the first population was considered as the objective population (population under positive selection) and the second one was the reference population (non-selected population). Thus, the population pairs considered were: CEU - YRI, YRI - CEU, CEU - CHB + JTP, CHB + JTP - CEU, CHB + JTP - YRI and YRI - CHB + JTP. A previous study that evaluated the performance of several genome scan methods [27] shows that the FDR of XPCLR increases substantially once the SNP reaches fixation. Therefore, we only used SNPs that were polymorphic in both populations in order to minimize false positives. Thus, there were 2,179,305 SNPs for population pairs including CEU and CHB + JTP, 2,203,610 SNPs for those that considered CEU and YRI and 2,118,211 SNPs for pairs including YRI and CHB + JTP. In principle, since humans originate in Africa, the YRI population should be considered as the reference population when looking for signatures of positive selection due to changed environmental conditions. However, we also carried out analyses with YRI as the objective population, as variants that were positively selected in Africa may be released from selective pressure in newly colonized habitats (Europe and Asia). We evaluated grid points every 200 bp along the genome and evaluated a window size of 50 SNPs around each point. We normalized the SNP scores across the whole genome such that they have zero mean and unit variance. This was performed separately for each of the pairwise comparisons conducted in the dataset.

#### iHS analyses

iHS scores for the three populations were derived for 1,131,343 SNPs for CEU, 1,264,811 for YRI and 976,006 for CHB + JTP. iHS is based on the decay in Extended Haplotype Homozygosity (EHH) to the right and left of a focal SNP, which is measured by the integrated Haplotype Homozygosity (iHH). More precisely, the EHH is calculated for all SNPs to the right and left of the focal SNP until EHH is equal to or larger than 0.05. However, if the focal SNP is close to the end of the chromosome or the start of a gap >200 kb, there may be no SNP for which EHH reaches or is below that value, in which case the iHS statistic cannot be calculated.

iHS performs better when the frequency of the selected allele is low (~0.1 and ~0.3) [27]. As in the case of the XPCLR analyses, iHS scores were also normalized. More details about the genome scan methods are given in Additional file 1: Text 1.

The results of the two genome-scan methods (XPCLR and iHS) for all the chromosomes in the HapMap Phase II database are available under request.

### Gene set enrichment analysis (GSEA)

After we calculated the selection scores (from XPCLR and iHS) for each of the SNPs in the dataset for all the different populations, we performed the GSEA. We used Daub et al. [19] approach and Gowinda to detect significant gene sets (more details are presented in Additional file 1: Text 2). This procedure was performed twice, using a) the XPCLR scores and b) the iHS scores.

#### Gene data

We downloaded the human protein coding gene data from the Entrez NCBI database from assembly GRCh38 on May 5/2014 and we extracted the start and the end position of 27,121 genes. For all genes that possess more than one reference sequence, we took the utmost start and end position of each of them (longest isoform).

#### Assignment of SNPs to genes

To match the SNP positions from assembly NCBI36 to gene positions (start/end) from assembly GRCh38, we converted the SNP positions from assembly NCBI36 to assembly GRCh38 using two tools: a) Remap tool from NCBI [28] and b) the liftOver tool from UCSC [29]. Conversion with the Remap tool was made on 5/3/2014 and conversion with liftOver on 10/3/2014. The SNPs that were mapped to the same location with both tools were kept for the final enrichment analysis.

#### Gene sets

We downloaded all the human gene sets from the Biosystems database [30] on 6/5/2014. The NCBI Biosystem database includes gene sets from KEGG, REACTOME, WikiPathways, Pathway Interaction Database (PID) and BIOCYC. We discarded gene sets that contain less than 10 genes [31]. To avoid redundancy due to duplicated gene sets, we merged gene sets with a similarity larger than 95 %. After these corrections, we were left with 928 gene sets out of the 2682 initial ones to continue the analysis.

#### Gene set enrichment analyses (GSEA) methods

Both Daub et al. [19] approach and Gowinda calculate the significance of gene sets based on permutation tests. They avoid biases due to differences in the length of genes and the overlap of the genes between gene sets. Nevertheless, the two methodologies are expected to give different results as they differ in several aspects, the most important being in the calculation of the gene set selection scores. Daub et al. [19] uses the selection scores from all SNPs in the analysis, estimating the sum of selection scores of all the genes in the gene set. On the other hand, Gowinda focuses only on the selection scores of the candidate SNPs, which are those that are significant for the chosen cut-off significance threshold (usually the 1- or 5-percentile of the total number of SNPs used in the genome scan). Other important differences include the permutation procedure, the correction of overlapping genes, and the correction for multiple testing. More details about the two GSEA approaches are given in Additional file 1: Text 2. To determine the significance in the real dataset, we used a q-value smaller or equal to 0.09 for both GSEA approaches.

#### Gene enrichment analysis for metabolic syndrome

To evaluate more in detail if the “Thrifty genotype hypothesis” can help explain the high incidence of diabetes 2, obesity, and metabolic syndrome in modern human populations we identified all genes that have been associated with these diseases using the Bio4j tool and the STRING database (see below). We then determined whether or not these genes were enriched for signatures of positive selection using the results of the XPCLR and iHS genome scans. A threshold of 1 % was used to determine significance of the gene scores.

##### Bio4j

Using the Bio4j tool [32], a graph-based platform that integrates semantically biological data from 6 different databases (Uniprot KB –SwissProt + Trembl, Gene Ontology, UniRef (50,90,100), NCBI Taxonomy, and Expasy Enzyme DB), we identified a total of 683 genes associated with obesity, diabetes 2 and metabolic syndrome. More details for Bio4j analysis are available in the Additional file 1: Text 3.1.

##### STRING database

We also used the Search Tool for the Retrieval of Interacting Genes (STRING) database [33] to find genes that interact with the candidate genes identified using Bio4j. The Protein-Protein Interaction (PPI) networks can reveal additional genes associated with metabolic syndrome, which could then be evaluated for signatures of positive selection. More details for STRING analysis are given in the Additional file 1: Text 3.2.

## Results and Discussion

In this study, we use the Cross Population Composite Likelihood Ratio (XPCLR) [20] and the integrated Haplotype Score (iHS) [21] to uncover the broadest range of positive selection signatures in the human genome. Numerous gene sets were identified to be involved in local adaptation but we focus on those related to immune responses and metabolic syndrome.

**Fig. 1:**
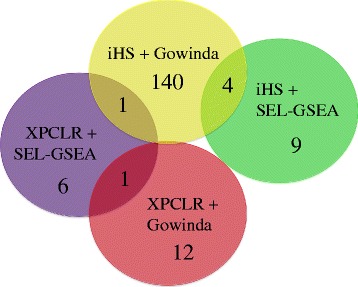
Illustration of the 167 gene sets that were detected with the four combinations of the genome scan and GSEA methods. Each circle shows the methods that were used with the number of candidate gene sets. The overlap among them is also shown

We found a total 161 gene sets enriched for signals of positive selection; 15 identified by Daub et al. [19] approach and 152 by Gowinda, with six that were identified by both. Figure 1 summarizes the results of the four GSEAs and the common gene sets that were found among them. An interesting observation is the large number of significant gene sets based on the iHS scores and Gowinda’s enrichment analysis (140). Using XPCLR, we performed six pairwise analyses of the three populations (Europeans, Chinese/Japanese, Yoruba) to investigate all the possible selection signals.

When iHS scores were used as input of the enrichment analysis, we found no significant gene sets for the CHB + JTP population with Gowinda and only one with Daub et al. [19] approach. In contrast, we identified a total of 140 different significant gene sets for the YRI population. Finally, the number of enriched gene sets obtained for the CEU population was four only (Table 1 and Additional file 2: Tables S1 and S2).

**Table 1:** Summary of the results of the enrichment analysis using iHS scores as the baseline

Population	GSEA: Daub et al. [19] (# of gene sets)	GSEA: GOWINDA (# of gene sets)	Total # of pathways for each population, and the in common pathways between the two GSEA approaches
CEU	3	2	5 of which 1 in common (Cell Cycle, Mitotic)
YRI	7	140	147 of which 3 in common (DNA Repair, Spliceosome, Cell Cycle, Mitotic)
CHB + JTP	1	0	1
Total # of pathways for each GSEA approach, and the in common pathways among populations	11 of which 2 in common (Cell Cycle, Mitotic, Olfactory Signaling Pathway)	142 of which 2 in common (Dna Repair, Cell Cycle, Mitotic)	152 of which 5 in common (Cell Cycle Mitotic, DNA Repair, Spliceosome, Olfactory Signaling Pathway, G2/M checkpoints)

When XPCLR scores were used as input for the enrichment analysis, we identified a total of 17 significant gene sets across all population pairs and enrichment analyses approaches (Table 2; Additional file 2: Tables S3 and S4). Using Daub et al. [19] approach, we found no significant gene sets for the population pairs CEU-CHB + JTP and YRI-CEU, while with Gowinda, we found no significant gene sets for the CEU-YRI population pair. Interestingly, when YRI was the objective population and CHB + JTP was the reference one, we detected seven gene sets, more than in the comparisons where YRI was the reference. We obtained similar results when YRI was the objective population and CEU was the reference, but only when using Gowinda.

**Table 2:** Summary of the results of the enrichment analysis using XPCLR scores as the baseline

Population pair (Objective-Reference)	GSEA: Daub et al. [19] (# of gene sets)	GSEA: GOWINDA (# of gene sets)	Total # of pathways for each population, and the in common pathways between the two GSEA approaches
CEU-YRI	3	0	3
YRI- CEU	0	2	2
YRI- CHB + JTP	1	7	8 of which 1 in common (Cell surface interactions at the vascular wall)
CHB + JTP-YRI	1	1	2
CEU- CHB + JTP	0	1	1
CHB + JTP-CEU	1	1	2
Total # of pathways for each GSEA	6	12	18 of which 1 in common (Cell surface interactions at the vascular wall)

The population on the left is considered to be the objective one, while the one on the right the reference one under all circumstances. Threshold is set to <0.09

Overall, these results suggest that selective pressures are much more important in Africa than in European and Asian populations. We posit that many of the enriched gene sets were under selection in Africa but were released from selection in the newly colonized habitats (Europe and Asia).

In what follows, we first present the results of the GSEAs based on XPCLR and then the results of analyses based on iHS mainly for the Yoruba population. We also explore candidates of positive selection that could be related to metabolism and therefore have been involved in the evolution of diseases such as diabetes type 2, obesity and metabolic syndrome.

### Infectious diseases

As individuals migrated to new environments (e.g. climate, diet), they were forced to adapt, and natural selection acted to favor variants that were advantageous both in pathogens and hosts. Both environmental factors and pathogen load must have had a great impact on the human genetic variation and infectious disease susceptibility. Based on all the analyses carried out, we identify a total 35 gene sets related to immune protection and host defense interactions; seven of them were detected with XPCLR and 28 with iHS (details about the populations that the gene sets were detected are presented in Additional file 2: Table S5).

### XPCLR-based GSEA

When Africa was compared with the Asian population (YRI: objective population), we observed increased selection pressures on host-defense mechanisms that we describe below in more detail (Additional file 2: Table S5).

Of primary importance is the *Cell surface interactions at the vascular wall* gene set, which was detected with both GSEA approaches. Platelets and leukocytes interact with the endothelium as a protective response of the host to infection by bacteria, pathogens or injury. Platelets can also enhance their activation through the generation of *signals by the alphaIIbbeta3 integrin gene set* [34], also significant in our analysis. Key regulator of host defense is the *T-Cell-Receptor (TCR) gene set,* whose main function is the induction of responses to the cell nucleus to isolate and destroy the malignant cells [35, 36]. Also significant was the *chemokine gene set,* which is directly involved in both innate and adaptive immune responses, as secreted chemokines and their receptors mediate leukocyte recruitment in the site of infection [37]. Xu et al. (2014) suggest that CXCRs, main receptors of the chemokine subfamily CXC, are also enriched for positive selection [38].

Although four immune mediated gene sets were detected in the pairwise comparison between YRI-CHB + JTP, in the opposite comparison (CHB + JTP-YRI) we detected only one, the *apoptotic* gene set, involved in programmed cell death. The dysfunction of genes involved in apoptosis can lead to inflammatory diseases and cancer [39–42]. da Fonseca et al. (2010) shows that positive selection signals on the apoptotic genes in mammals are mainly the result of evolutionary forces between host and pathogens [43].

These observations overall suggest that gene sets related to host defense interactions in the immune system were under selective pressures in Africa. However, they were released from such pressures once humans migrated to Asia, presumably because many of the pathogens (bacteria, viruses and other infectious pathogens) were absent in the newly colonized continent.

### iHS-based GSEA

Interestingly, numerous gene sets (147) were detected as enriched for signatures of positive selection in the YRI population using iHS. Here we mainly focus on the immune- and host defense- related ones.

Ten gene sets associated with different phases of *Human Immunodeficiency Virus* (HIV) and HIV-1 infection were detected in the African population (Additional file 2: Table S5). HIV is among the most threatening infectious diseases in Africa and its prevalence accounts for 71 % of HIV worldwide [44]. The genetic diversity in HIV patients is believed to be the result of the virus evolution in relation to the human immune system [45, 46]. Positive selection was also detected in previous studies on the human genome of HIV patients [47] as well as on HIV genome sequences [45, 48, 49], indicating selective pressures on both the virus and the host.

Three other significant gene sets, the *Salmonella*, *Shigellosis*, and *Helicobacter pylori (H. pylori) infection gene sets* are clearly involved in the defense against pathogens. These infections are usually caused by environmental contamination (e.g. food or water) [50–52]. Bacteria continuously evolve to find new strategies to deceive the host barriers [53]. Positive selection on the specific bacteria strains [54–56] is consistent with an evolutionary arm race between bacteria and the human host immune system. Salmonella and Shigella use the same mechanism, known as the trigger model, to invade organisms [57], whereas *H. pylo*ri adheres to and reproduces in epithelial cells [58]. Interestingly, we also detected selection pressures on the *invasion of bacteria through epithelial cells* gene set. Epithelial cells are involved in antimicrobial host immune defense and induce inflammatory responses to prevent the pathogens from invading [59].

The *Toll-Like-Receptors (10 different cascades were significant)* and *RIG-1 receptors (2 different significant pathways)* play a critical role to introduce innate immune responses. They are associated with the recognition and defense of pathogenic microorganisms, viruses and bacteria [60, 61]. Two *Interleukin gene sets*, a group of cytokines that secret signals to regulate the immune response, were also identified as candidates of positive selection.

Overall, our results indicate that selective pressures on host-defense mechanisms exerted by pathogens in Africa became much less important once humans colonized other continents because of dramatic changes in the environment experienced by colonizers. In agreement with previous observations, selection is associated with protective immunity (e.g. *TLR*s- *RIG*-1 receptors, interleukin) against infectious diseases (e.g. bacterial infections, HIV) [19, 61–66].

### Obesity – metabolic syndrome – diabetes

Obesity, diabetes and metabolic syndrome are chronic diseases that have been thoroughly studied. A large effort has been made to find variants, genes and gene sets that contribute to their pathogenesis. Although many studies have identified multiple genetic factors, few have examined whether or not they are enriched for signatures of positive selection. Past selective pressures could help explain the high prevalence of metabolic syndrome in modern human populations [67–70]. More precisely, changes in environmental conditions, nutrition, or lifestyle may induce radical changes in selective patterns; genes initially subject to positive selection in ancient humans may become deleterious under the environmental conditions experienced by modern human societies. Potential examples are the insulin receptor substrate-1 (*IRS1*) and *TCF7L2* genes that have already been found to be under positive selection [71, 72]. Nevertheless, given the likely polygenic architecture of metabolic syndrome, it is important to carry out gene set enrichment analyses in order to identify new candidate gene sets. In our study, we found two gene sets that could be associated with metabolic syndrome; the *Glycolysis and gluconeogenesis* and the *Signal Attenuation gene sets.*

The *Glycolysis and gluconeogenesis gene set* was detected with XPCLR and Gowinda when the European was the objective population and the Asian was the reference population. In principle, glycolysis and gluconeogenesis are two metabolic processes that have opposite effects. Glycolysis degrades glucose in periods of fasting, diet or intense exercise and gluconeogenesis produces glucose in periods of feeding. The role of glycolysis and gluconeogenesis gene set is well defined in the pathogenesis of diabetes. Glycolysis controls the hepatic glucose production [73], which causes hyperglycemia, a symptom of diabetes. It is also involved in cases of insulin resistance and deficiency [74], which is usually observed in type 2 diabetes [75]. Felig et al. (1974) also indicates that glucose precursors are increased in obese population [76], although the clear role of this gene set in obesity is still controversial [77]. A few studies have already investigated the possibility of glycolysis and gluconeogenesis gene set as targets for diabetes therapies [78–80].

Our result are consistent with the hypothesis that in the past, the glycolysis and gluconeogenesis gene set was subject to positive selective pressures associated with adaptation to potentially long fasting periods. But it then may have become maladaptive due to extreme changes in the environment experienced by humans in modern societies. We suggest that these results could be explained by the fact that recent negative selection should be pronounced in Europeans but not in Asians presumably because of differences in diet and lifestyle (e.g. food, increased sedentarily and limited exercise). For example, Europeans tend to consume more meat than Asians [81], and it has been shown that increased consumption of meat increases the incidence of diabetes 2 [82].

We also detected the *signal attenuation gene set* in the European population, which includes 15 insulin related genes. This gene set was identified in the comparison with the Africans (CEU-YRI pair) using XPCLR and Daub et al. [19] approach. We note that the significance of this gene set may be due mostly to the top-scoring outlier gene, *DOK1*, which plays an important role in insulin receptor binding [83]. Nevertheless, insulin related genes closely interact with *DOK1* [84], according to the STRING database (Additional file 1: Figure S2). Among them, the *IRS1* and *IRS2* genes play an important role in the pathogenesis of diabetes and obesity [85–88]. Our analysis did not detect the IRS- genes presumably because of the stringent cut- off level that we used, however, a previous study has shown that IRS1 is under positive selection [72].

Lastly, we identified the *autoimmune thyroid disease* in the pairwise comparison between Europeans (objective) and Yoruba, which is an inflammatory disease in the thyroid gland where blood cells and antibodies attack healthy tissue. Interestingly, it has also been associated with the pathogenesis of most types of diabetes [89] and its prevalence shows a ~4 % increase in diabetic populations compared to non-diabetic. Our findings agree with Jin et al. (2012) [90], that also detected the auto-immune thyroid disease and *allograft rejection* gene sets enriched for positive selection in populations from Sub-Saharan Africa and North America using an Fst-based genome scan method.

Having identified gene sets associated with metabolic syndrome and enriched for signatures of positive selection, we next investigated if individual genes potentially associated with this disease, based on current knowledge, were also enriched for signatures of positive selection. To this end we used Bio4j to extract the genes that are associated to metabolic syndrome and then used STRING to identify genes that interact closely with them.

Using this approach, we identified 23 genes associated with metabolic syndrome and enriched for signatures of positive selection; 17 of them were detected with the XPCLR-based analysis and six other were detected with iHS (Additional file 2: Tables S6 and S7 respectively). Ten of them (*SLC19A2, SLC2A10, UCP2, SLC27A4, BLK, ESP15, EGFR, SOCS1, TSC*, and *PTH*) have already been suggested to be under positive selection [15, 91–101]. That leaves us with 13 new genes associated directly (six) and indirectly (seven) with obesity, diabetes or metabolic syndrome and also subject to natural selection. Interestingly, the *KDM3A* (or *Jhdm2a*) gene also enriched for positive selection is a key regulator for obesity and metabolic syndrome in mice [102, 103], but it has not been studied thoroughly in human populations. Nine of the 23 genes play a potential risk role in the development of diabetes, obesity or metabolic syndrome. Five of them (*GATA6, CP, LARS2, MRAP2* and *SGIP1*) belong to the 13 novel genes that were identified in the current analyses. Four in total have a potential protective effect, with the *PIK3CB* gene to be a novel candidate for positive selection, detected in the comparison of Asians (objective) with Europeans (reference). *PIK3CB* was previously shown to be associated with protection against insulin resistance [104]. The rest of the genes have an indirect association without clear evidence for their direct implication in metabolic syndrome (Additional file 2: Tables S6 and S7).

**Fig. 2:**
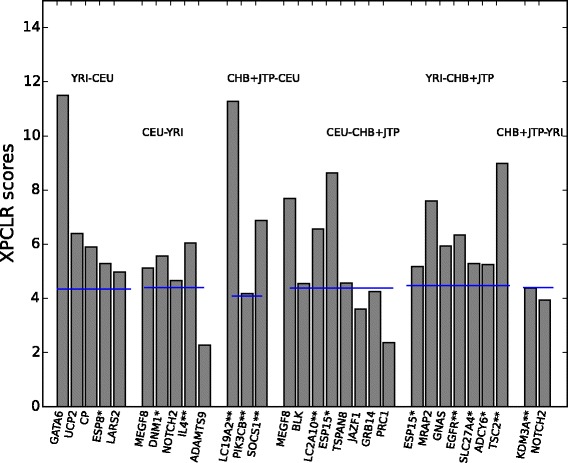
Strength of positive selection (XPCLR scores in the y-axis) on genes that are associated with metabolic syndrome. The blue line illustrates the 1 % cut off for each pair. The population pairs where these genes were detected are shown on the top. The first population is always the objective and the second one is the reference. Genes with no star symbol were suggested to be susceptible to metabolic syndrome, with one star, they need further investigation for their association with metabolic syndrome and with two stars, they might offer protection against metabolic syndrome

Comparing our results with two previous analyses on diabetes [68, 69], we found that *TSPAN8* and *NOTCH2* genes were also detected in our analyses, while some others (*ADAMTS9, JAZF1, GRB14* and *PRC1*) genes were just below the threshold we used. Figure 2 presents *the* genes that show evidence of positive selection, and are risk or protective factors for metabolic syndrome. Surprisingly, we do not detect the *TCF7L2* gene, unlike many studies [71, 105]. Nevertheless, the *TBL1XR1* and *CUL1* genes that closely interact with *TCF7L2* gene (Additional file 1: Figure S3; derived from STRING database) are under positive selection in our analysis.

## Conclusions

In this study, we were particularly interested in uncovering selection signatures due to changed environmental conditions found by expanding human populations as they left the African continent. We hypothesized that such changes can influence gene sets involved in immune response to pathogens or associated with diseases such as diabetes, obesity and metabolic syndrome, which could be connected with changes in life-style or diet.

It is widely accepted that after the first exodus from Africa, humans confronted changed environmental conditions and had to face new pathogens [106, 107]. Therefore, migrants were likely to encounter new selective pressures. The large number of positively selected gene sets that we found in the African population, mainly using the iHS genome scan method, suggests that the most common scenario involved a release from selective pressures present in the African continent but absent in Europe and Asia. Indeed, our results show that selective pressures on immune related- and host-defense gene sets are detected in Africa but not in Eurasia. This result is consistent with the idea that pathogens may be more abundant in Africa than in other continents [108].

It is important to note that the signature of selection identified by iHS (long stretches of homozygosity) is similar to that left by purifying selection acting on conserved regions [109]. In this regard, we observed that gene sets uncovered by both Daub et al. [19] approach and Gowinda when using the iHS selection scores (*spliceosome, cell cycle, mitotic and DNA repair*) are conserved across several species. The distribution of the iHS scores of some of the conserved pathways (Additional file 1: Figure S3), does not give a clear pattern whether selection in the pathways are mainly driven by selection on the ancestral or the derived allele. Thus, although iHS is widely used to detect positive selection, we posit that several of the gene sets identified by iHS might in fact be subject to purifying selection instead of local adaptation. However, according to previous studies [109, 110], this happens mainly in non-coding regions, unlike the gene sets detected here that are in coding regions.

We investigated thoroughly the gene sets and the genes that could be associated with metabolic syndrome, mainly diabetes and obesity, whose prevalence increased in recent years. We examine diabetes and obesity as a single entity because they are intimately associated. More specifically, obesity is considered one of the most important factors in the pathogenesis of diabetes [111]. Our results suggest that metabolic syndrome could in part be explained by the thrifty genotype hypothesis. Even though, there is a controversy on whether or not insulin resistance increased metabolic efficiency in the past [112], our result is in agreement with the reduced selection pressure on the Glycolysis and Gluconeogenesis gene set that was observed in the Asian population (see above). The process of gluconeogenesis is controlled by insulin hormone but in the case of insulin resistance, it fails to stop glucose production [74]. Therefore, there might be an association between the deficiency of the insulin receptor and increased levels of nutrients that promoted fat storage in early humans. Lastly, two potentially protective genes, *EGFR* and *SOCS*1, were identified with iHS, which suggests that these genes may be undergoing recent selective sweeps associated with the high prevalence of metabolic syndrome in modern humans.

Our analyses uncovered several new gene sets with immune- and glucose- related functions that may be subject to positive selection. Moreover, it presents a substantial number of novel genes associated with diabetes and obesity that are enriched for signatures of positive selection. Overall, our study brings new insight into the emergence and evolutionary history of infectious diseases and metabolic syndrome. In the era of unlimited public data resources and cutting-edge approaches to analyze them, understanding the way that polygenic selection has shaped the human genetic diversity could lead towards better prevention and treatment of these diseases.

## Abbreviations

CEU, Europeans; CHB + JTP, Han Chinese from Beijing, China and Japanese from Tokyo; GSEA, gene set enrichment analysis; iHS, integrated haplotype score [20]; XPCLR, Cross Population Composite Likelihood Ratio (XPCLR) [23]; YRI, Yoruba from Ibadan, Nigeria.

## Additional files

Additional file 1: Supplementary Material. (DOCX 1057 kb)

Additional file 2: Table S1. Significant gene sets found using iHS scores and Daub et al. [19] approach. Threshold is set to <0.09. **Table S2.** Significant gene sets found using iHS scores and Gowinda approach. Threshold is set to <0.09. **Table S3.** Significant gene sets found using XPCLR scores and Daub et al. [19] approach. Threshold is set to <0.09. For all the population pairs, the first population is the objective one and the second, the reference. **Table S4.** Significant gene sets found using XPCLR scores and Gowinda approach. Threshold is set to 0.09. For all the population pairs, the first population is the objective one and the second, the reference. **Table S5.** Immunity related gene sets detected with the GSEA approaches. Q-value threshold is set to <0.09. **Table S6.** 17 significant genes related to obesity, diabetes and metabolic syndrome that were found to be under positive selection with XPCLR and iHS analysis using the list of genes derived from Bio4j. Some of them (indicated with *) have been detected in previous studies to be under positive selection, too. The threshold was calculated based on the 1 % cut off level. Genes are categorized in groups of potential risk factors, potential protective and indirect associations. **Table S7.** Significant genes related to obesity, diabetes and metabolic syndrome that we found to be under positive selection from XPCLR and iHS analysis using the Protein-Protein Interaction (PPI) networks from the STRING database. Five of them (indicated with *) have been detected in previous studies to be under positive selection. (XLSX 29 kb)
