# Supplementary material for: Changes in selective pressures associated with human population expansion may explain metabolic and immune related pathways enriched for signatures of positive selection
Source: BMC Genomics. 2016 Jul 21;17:504. doi: 10.1186/s12864-016-2783-2 (PMC4955149; doi:10.1186/s12864-016-2783-2)
Supplement: Supplementary file 1 — Supplementary Material. (DOCX 1057 kb) [file 12864_2016_2783_MOESM1_ESM.docx]

Supplementary Materials

**Changes in selective pressures associated with human population expansion may explain metabolic and immune related pathways enriched for signatures of positive selection.**

**Alexandra I. Vatsiou^*1,2,3^, Eric Bazin^1^, Oscar Gaggiotti^1,2^**

^1^Laboratoire d'Ecologie Alpine, University Joseph Fourier, Grenoble, France

^2^Scottish Oceans Institute, East Sands, University of St Andrews, St Andrews,

KY16 8LB, UK

^3^Oh no sequences! Research group, Era7Bioinformatics, Granada, Spain

^*^Corresponding author: E-mail: [alex.vatsiou@gmail.com](mailto:alex.vatsiou@gmail.com)

keywords: positive selection, enrichment analysis, gene sets, metabolic syndrome

**Text 1: Genome Scan Methods**

- 1. ***XPCLR method***

*Cross Population Composite Likelihood Ratio (XPCLR*) [1] is a two-populations test that considers an objective population (under positive selection) and the reference population (under neutrality). It focuses on multilocus allele frequency differentiation between those two populations to identify regions were changes in allele frequency are unlikely to be due to random genetic drift. The method is based on an expression for the distortion of frequency at a neutral allele in the vicinity of a selected one in the population under selection. The method detects the selected allele by conditioning on the allele frequency in a second population free of selection. A composite likelihood approach is used to apply the previous model to a region (a window comprising multiple SNPs) so as to obtain a multilocus measure of genetic differentiation for each region. XPCLR detects selective sweeps where the favored allele has intermediate (~ >0.3) to high frequencies.

- 1. ***iHS method***

*Integrated Haplotype Score (iHS)* [2] is an extension of the Extended Haplotype Homozygosity (EHH) test of Sabeti et al. (2002) [3]. EHH is based on the decay of EHH with distance from a core SNP (SNP of interest). The decay is much slower under selection than under neutrality due to the linkage disequilibrium that is created. Thus, the method is based on the calculation of iHH (integrated Haplotype Homozygosity), the integral of the observed decay of EHH away from the specified core SNP until it reaches 0.05, for both the ancestral (A) and derived (D) alleles. The iHS score is then the logarithm of the ratio iHH_A_/iHH_D_.

**Text 2: Gene Set Enrichment Analysis**

**2.1 Daub et al. (2013) approach**

*Assignment of SNPs to genes*

Daub et al. (2013) uses all the SNPs (candidates and non-candidate for positive selection) to make inferences about the gene sets. To acquire one selection score for each gene in the dataset, firstly all SNPs were assigned to genes if they were located within the gene transcript or within 50kb upstream or downstream of the start/end of the gene. Then, we took as representative selection score per gene, the highest of the SNP scores assigned to the gene. To further account for the possible bias longer genes be assigned a larger number of SNPs than shorter ones, we made a further normalization. We grouped genes to bins according to the number of SNPs they have. We then normalized the score of each gene based on the distribution of the bin. In what follows, we refer to the gene score as g(s)

$g\left( s \right)=\frac{{g\left( s \right)-mean(g(s))}_{bin}}{{std(g(s))}_{bin}}$ (1)

*SUMSTAT*

To examine if gene sets are enriched for signatures of selection, we calculated their scores [4] by simply summing the g(s) scores of all genes in the gene set. We will refer to this statistic as SUMSTAT. To evaluate significance, we inferred empirical p-values for each gene set, by comparing each of the gene-set scores to an empirical null distribution of SUMSTAT scores. To acquire the null distribution, we draw 10000 random gene sets for each of the different lengths of the gene sets in the dataset. Then, we also acquired the q-values for each gene set using the package q-value in R [5]. Gene sets with q-value<0.09 where considered enriched for positive selection.

*Pruning*

To avoid bias due to the large number of genes that are shared among the different gene sets, we used a pruning method involving the following steps (let LGS be the List of Gene Sets) following Daub et al. (2013):

1) Rank all the gene sets in LGS according to their p-value (from lowest to highest P value).

2) Remove the first gene set S from LGS and store it in a new list LGS1.

3) Remove the genes in S from the remaining gene sets in LGS and from the gene list.

 4) Remove all gene sets in LGS for which their length is smaller than 10.

5) If LGS contains more than one gene set:

5a) Calculate the SUMSTAT values for the trimmed gene sets that have remained in LGS.

5b) Calculate the empirical p-values for the current trimmed gene sets. As described previously, we determine significance by sampling random genes using every time the current gene list. We draw 10000 random gene sets for each of the lengths of the gene set list.

5c) Rank the sets in LGS according to their p-value and go back to step 2

6) If LGS contains one gene set, stop the pruning procedure and calculate the q-values of the trimmed gene sets in LGS1, as described below.

*Empirical correction for multiple testing*

After correcting the gene sets for overlapping genes, we used a randomization procedure to calculate the q-values for the trimmed gene sets. We produce through permutations the expected distribution of the p-values, and we produced a map of p-values (P) to a FDR(P) [6] as follows:

$$\hat{FDR}\left( P \right)= \frac{m*P*\pi_{0}}{S\left( P \right)} (2)$$

where m is the total number of gene sets after pruning, P is the current threshold, π_0_ is the total number of true null hypothesis and S(P) the number of rejected null hypothesis (number of gene sets in the observed data that have a p-value greater or equal to P).

The number of true null hypotheses (π_0_) was approximated using a histogram-based method [7-8], which simply compares the observed with the expected distribution of p-values. In order to obtain the expected distribution, we permuted the g(s) in the whole gene list and we repeated the Daub et al. (2013) approach with the pruning 50 times. Then, we split the p-values that were obtained after the pruning of each repetition in bins. Then, we compare the distribution of each p-value bin between the expected and the observed data by calculating the mean proportion of gene sets that belong to each bin. To calculate the approximate π_0_, we identify the first bin i for which the expected distribution exceeds the observed one with corresponding p-value x and we calculated the approximated π_0_, as follows:

$$m0= \frac{\sum_{i}^{J} n_{i}}{1-x}{(3) and \pi}_{0}= \frac{m0}{m} (4)$$

where i is the bin (index) of p-value x, J is the bin with p-value 1 and n is the observed proportion of p-values in bin i [9].

# *2.2 Gowinda*

As a second GSEA approach, we used Gowinda [10] to carry out separate enrichment analyses based on the XPCLR and iHS genome scan results. Gowinda takes as an input four files: 1) the list of all SNPs in the dataset, 2) the gene list, 3) the mapping of genes to gene sets and 4) the selection scores of SNPs that tested positive (candidate SNPs). In our case, we considered candidate SNPs, the SNPs with a significant XPCLR and iHS score that belong in the 1% cut-off considering the whole genome. The results were obtained after running 1000000 permutations. We conduct this analysis under the mode *–gene,* which assumes all the SNPs in the gene are completely linked.

**Text 3: Genes in metabolic syndrome**

***3.1 Bio4j analysis***

Bio4j is a graph-based platform that integrates the big data from six different databases ([**Uniprot KB**](http://www.uniprot.org/) (SwissProt + Trembl), [**Gene Ontology**](http://www.geneontology.org/) (GO), [**UniRef**](http://www.ebi.ac.uk/uniref/) (50,90,100), [**NCBI Taxonomy**](http://www.ncbi.nlm.nih.gov/Taxonomy/), and [**Expasy Enzyme DB**](http://enzyme.expasy.org/)) and it provides a more structured semantically level typed graph database [11]. Bio4j uses query languages that allow the users to semantically query the database about genes, proteins as well as the relationship between them and therefore access and extract the information needed.

In our case we perform a semantically guided analysis using all the available data resounces in Bio4j, using as key word the words “obesity OR metabolic syndrome OR diabetes”. We extracted in total 683 genes that could directly or indirectly be associated with obesity or metabolic syndrome or diabetes according to previous studies. Out of the 683 genes, we found a total of 18 genes to be under positive selection (Table SI5). 13 of them were detected with the XPCLR-based analysis and 4 with the iHS-based analysis. We used a threshold of 1% to determine significance.

***3.2 STRING analysis***

We extended our research about metabolic syndrome to Protein-Protein Interaction (PPI) level. It is well accepted that PPI can reveal information about target “hidden” genes that play an important role in therapies and in the identification of complex diseases. Several methods exist to conduct such an analysis, one of which is the Search Tool for the Retrieval of Interacting Genes (STRING) database [12]. We chose the STRING database because integrates an enormous amount of proteins and interactions (5 million proteins and >200 million interactions) [13]. The goal was to find interactions with genes that could be under positive selection and are associated with metabolic syndrome. STRING database takes as an input a list of genes and it finds the PPI among them and other genes. We created two PPI networks using the default parameters in STRING database: 1) with the insulin related genes that we found from the Signal transduction gene set and 2) with the positively selected genes detected by Bio4j. Our goal for these PPI networks is to observe their-in-between interactions and uncover further “interesting” genes that are under positive selection in our analysis.

When we used the positively selected genes detected by Bio4j (17 genes), with confidence 95% and a maximum of 500 interactions, only three of them (BLK, GNAS and PIK3CB) interacted with each other (Figure SI1). The final PPI network consisted of 42 interactions. 34 genes out of the 42 are also genes that are included in the significant pathways that we found. However, only *three (EGFR, PTH and ADCY6)* of those are significant for positive selection in the gene-level threshold.

When we used as an input, the 15 insulin-related genes (IRS1, IRS2, DOK1, GRB10, INS-IGF2, INS, INSR, MAPK3, MAPK1, CRK, GRB2, SOS1, SHC1, SHC3, SHC2) from the signal attenuation gene set, we defined the parameters of the STRING database for the network as follows: confidence to 99.6% and maximum number of interactions in the network to 500. In the end, we had a PPI network with a total of 82 interactions (Figure SI2). Seven of the genes included in the network (DOK1, ESP15, EGFR, SHC1, SOCS1, GRB2 and TSC2) are positively selected in our analysis. DOK1, SHC1 and GRB2 were used as an input, a fact that leaves as with 4 new candidates for positive selection (ESP15, EGFR, SOCS1 and TSC2) that could be associated with metabolic syndrome.

To summarize, STRING database revealed a total of six different genes *(ESP15, EGFR, SOCS1, TSC2, PTH and ADCY6)* to be enriched for positive selection and are associated with metabolic syndrome (Table SI6).

**Legends for Figures**

*Figure SI1*: PPI network that was created by STRING database using as input the positively selected genes that were detected by Bio4j.

*Figure SI2:* PPI network that was created by STRING database using as input the insulin related genes from the Signal attenuation gene set.

*Figure SI3:* Distribution of iHS scores for four conserved pathways a) the spliceosome in the YRI population b) spliceosome in the CEU population c) DNA repair gene set in the YRI population and d) Cell Cycle Mitotic gene set in the CEU population.

Figure 1


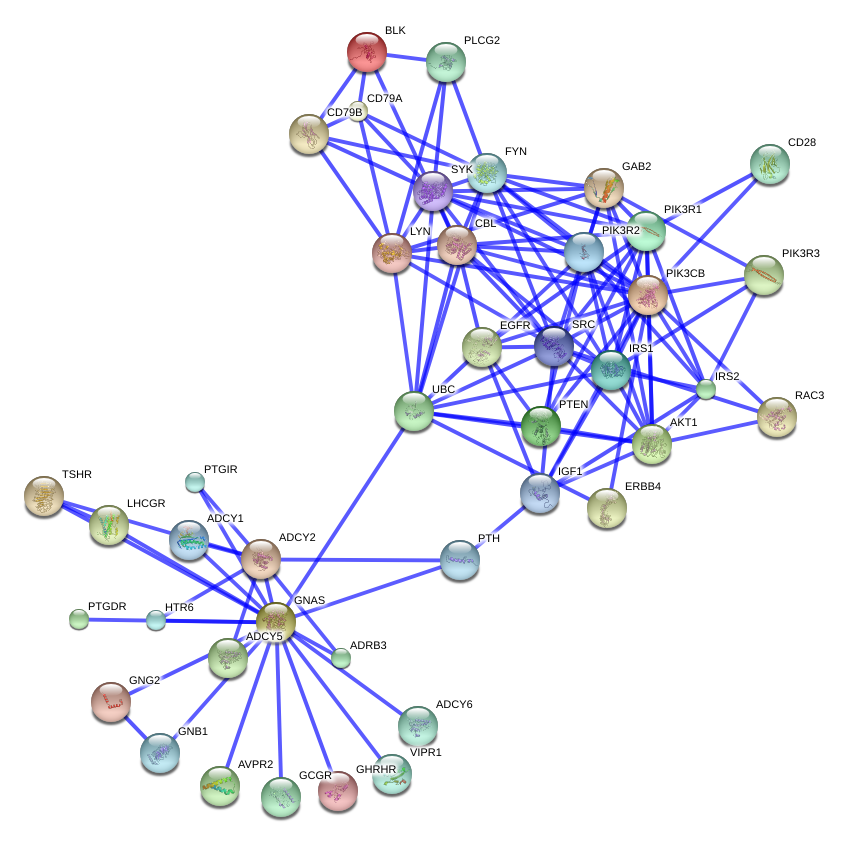


Figure 2


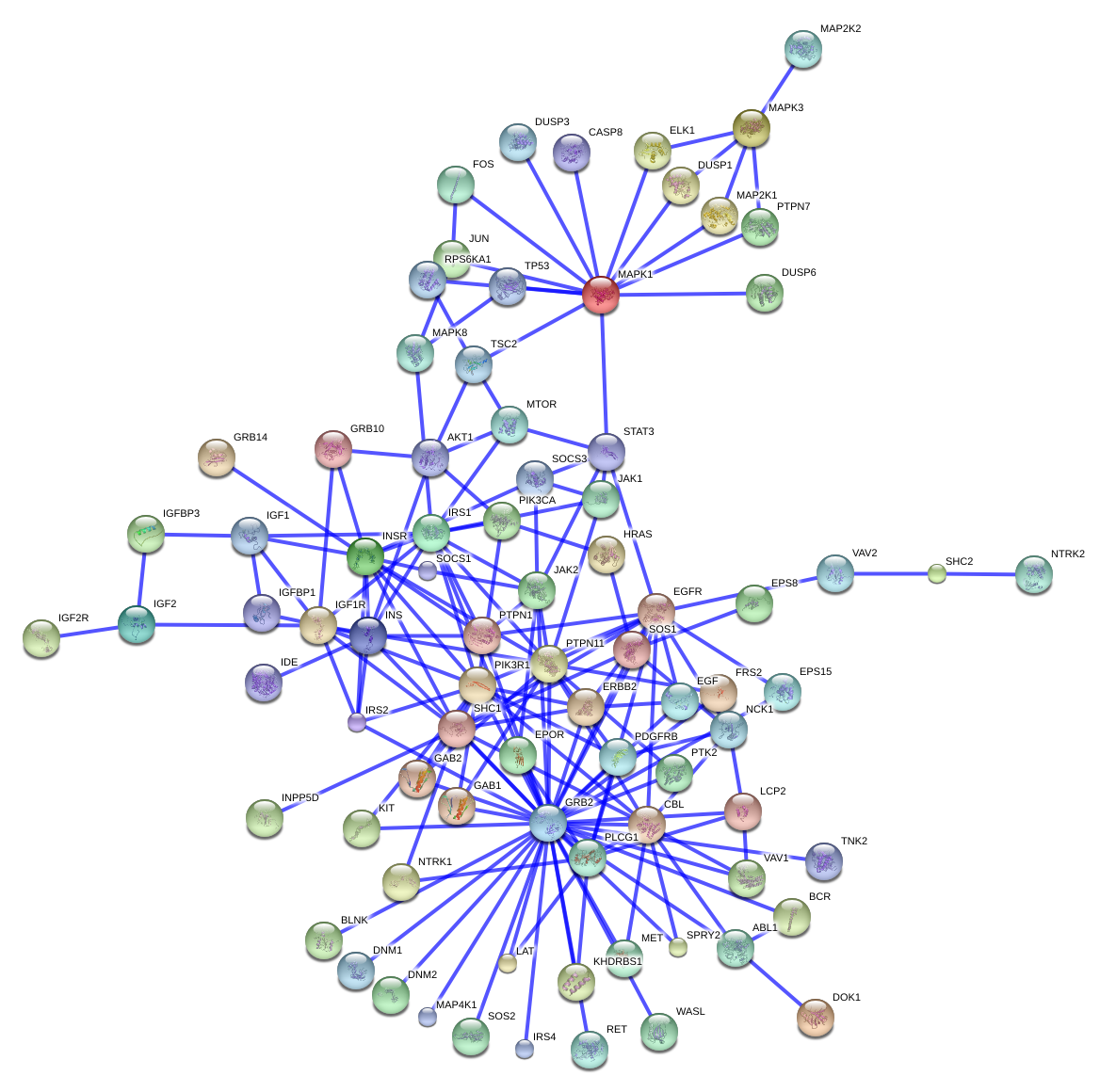


Figure 3

**References**

1. Chen H, Patterson N, and Reich D. Population differentiation as a test for selective sweeps. Genome research. 2010;20:393-402.
2. Voight BF, Kudaravalli S, Wen X, and Pritchard JK. A map of recent positive selection in the human genome. PLoS biology. 2006;4:e72.
3. Sabeti PC, Reich DE, Higgins JM, Levine HZ, Richter DJ, Schaffner SF, Gabriel SB, Platko JV, Patterson NJ, McDonald GJ*, et al*. Detecting recent positive selection in the human genome from haplotype structure. Nature*.* 2002;419:832-837.
4. Daub JT, Hofer T, Cutivet E, Dupanloup I, Quintana-Murci L, Robinson-Rechavi M, and Excoffier L. Evidence for polygenic adaptation to pathogens in the human genome. Molecular biology and evolution. 2013;30:1544-1558.
5. Storey JD, and Tibshirani R. Statistical significance for genomewide studies. Proceedings of the National Academy of Sciences of the United States of America. 2003;100:9440-9445.
6. Tibshirani JDSaR. Statistical significance for genomewide studies. PNAS. 2003;100,9440-9445.
7. Mosig MO, Lipkin E, Khutoreskaya G, Tchourzyna E, Soller M, and Friedmann A. A whole genome scan for quantitative trait loci affecting milk protein percentage in Israeli-Holstein cattle, by means of selective milk DNA pooling in a daughter design, using an adjusted false discovery rate criterion. Genetics. 2001;157:1683-1698.
8. Nettleton D, HJ, Caldo RA, Wise RP. Estimating the number of true null hypotheses from a histogram of p values. J Agric Biol Environ Stat. 2006:337-356.
9. Bancroft TJ. Estimating the number of true null hypotheses and the false discovery rate from multiple discrete non-uniform permutation p-values. Graduate Theses and Dissertations *Paper 10063*. 2009.
10. Kofler R, and Schlotterer C. Gowinda: unbiased analysis of gene set enrichment for genome-wide association studies. Bioinformatics; 2012;28: 2084-2085.
11. Pareja-Tobes P, Tobes R, Manrique M, Pareja E, and Pareja-Tobes E. Bio4j: a high-performance cloud-enabled graph-based data platform. 2015. [Database website: <http://bio4j.com/>].
12. Snel B, Lehmann G, Bork P, and Huynen MA. STRING: a web-server to retrieve and display the repeatedly occurring neighbourhood of a gene. Nucleic acids research. 2000*;*28:3442-3444. [Database website: <http://string-db.org/>].
13. Franceschini A, Szklarczyk D, Frankild S, Kuhn M, Simonovic M, Roth A, Lin J, Minguez P, Bork P, von Mering C*, et al*. STRING v9.1: protein-protein interaction networks, with increased coverage and integration. Nucleic acids research. 2013;41:D808-815.
